# Diabetic retinopathy as a potential risk factor for ptosis: A 13-year nationwide population-based cohort study in Taiwan

**DOI:** 10.3389/fepid.2023.1093064

**Published:** 2023-03-13

**Authors:** Chun-Ju Lin, Alan Y. Hsu, Peng-Tai Tien, Cheng-Hsien Chang, Chun-Ting Lai, Ning-Yi Hsia, Yu-Cih Yang, Henry Bair, Huan-Sheng Chen, Wen-Lu Chen, Yi-Yu Tsai

**Affiliations:** ^1^ Department of Ophthalmology, China Medical University Hospital, China Medical University, Taichung, Taiwan; ^2^ School of Medicine, College of Medicine, China Medical University, Taichung, Taiwan; ^3^ Department of Optometry, Asia University, Taichung, Taiwan; ^4^ Department of General Medicine, China Medical University Hospital, Taichung, Taiwan; ^5^ Graduate Institute of Clinical Medical Science, College of Medicine, China Medical University, Taichung, Taiwan; ^6^ Department of Ophthalmology, Changhua Hospital, Changhua, Taiwan; ^7^ Management Office for Health Data, China Medical University Hospital, Taichung, Taiwan; ^8^ College of Medicine, China Medical University, Taichung, Taiwan; ^9^ Byers Eye Institute, Stanford University School of Medicine, Stanford, CA, United States; ^10^ An-Shin Dialysis Center, NephroCare Ltd., Fresenius Medical Care, Taichung, Taiwan

**Keywords:** age, diabetic retinopathy, gender, hypertension, lipid disorders, ptosis, smoking

## Abstract

**Purpose:**

To determine the risk of ptosis among diabetic retinopathy (DR) patients.

**Methods:**

This is a population-based, retrospective, matched-cohort study where DR patients were recruited from the Taiwan National Health Insurance Research Database (NHIRD) to investigate the risk of developing ptosis. Preexisting co-factors of interest included smoking status and medical comorbidities of hyperlipidemia and hypertension. Statistical analysis was performed using *T*-test, Cox-proportional hazard ratios adjusted for comorbidities (aHR), Wilcoxon rank sum test, Kaplan–Meier estimators, and log rank tests.

**Results:**

Follow-up data of 9,494 patients with DR and 37,976 matched control cohort (non-DR) from 2000 to 2012 were analyzed. DR patients were found to have significantly increased risk of developing ptosis (adjusted hazard ratio (HR) [95% CI]: 2.76 [1.74–4.38], *p* < 0.001) when compared to the control cohort. From analysis in different strata, adult age and non-smokers were shown to have higher risk for ptosis development among DR patients. Furthermore, DR patients was also found to have increased risk of developing ptosis when compared to matched controls, regardless of whether they had medical comorbidities of lipid metabolism disorders or hypertension.

**Conclusions:**

In this large-scale study using real-world data, our results showed that DR patients were found to have increased risk of developing ptosis. Female gender, adult age, and non-smokers were also shown to increase the risk of ptosis among DR patients. This has implications towards the care of diabetic patients, complications such as ptosis should be properly screened for when encountering such patients. Before ptosis surgery, the possibility of underlying diabetes or DR should be also scrutinized and treated properly to avoid undesirable postoperative dissension.

## 1. Background

Diabetes mellitus (DM) is a multi-system condition that is increasingly recognized as an emerging global epidemic (1). At present, there are about 425 million diabetic patients globally, and this number is projected to hit 629 million by the year 2,045 (2). In terms of Taiwan, the prevalence of DM among the adult Taiwanese population was estimated to be around 11.8%. DM is associated with a number of complications including diabetic retinopathy (DR). DR is a type of a neuro-vascular complication secondary to DM and is a leading cause of blindness among people aged 20–64 years old (3). Common presentations of DR include blurry vision and floaters. Although DR usually presents bilaterally, its insidious onset often makes early diagnosis quite challenging. By the time it is clinically apparent, DR are often at an advanced clinical stage where irreversible damage to the eyes would have already occurred (4). In Taiwan, the Bureau of National Health Insurance (NHI) oversees the DR screening programs. Every country has their own variations, but in general, every national program is tasked with the implementation of set protocols that allows for the timely referral of DR patients to appropriate medical facilities in order to achieve early diagnosis of DR in the majority of diabetic patients. The primary goals of treating diabetic patients with DR is to improve their quality of life. One of the ways to achieve this is by preventing diabetic comorbidities commonly seen among DR patients. In a study by Davidov et al., complications such as peripheral vascular disease, coronary artery disease or cerebrovascular disease have been shown to be more frequent among patients with retinopathy (5). Few studies however have assessed for the complications of ptosis developing among DR patients specifically.

Ptosis is characterized by the drooping of the upper eyelid (6) and is another common ophthalmologic condition worldwide with an estimated 11.5% of adults in the United Kingdom over the age of 55 years old affected (7). Ptosis may be broadly subdivided into its various causes, which includes: myogenic, neurogenic, aponeurotic, mechanical or traumatic. Malposition of the levator muscle tendon is the most common cause of adult onset ptosis (8). Ptosis not only results in functional visual impairments, but can have aesthetic consequences for the patient as well. Although some studies have linked ptosis with DM - with the earliest dating back to 1965 (9), most of the studies so far have been limited to small power of retrospective nature (10–14). Thus, further research are needed to confirm the risk of ptosis complications among DR patients. Better understanding of the relationship between DR and the future risk of ptosis complications would allow clinicians to better care for patients with DR and DM alike.

The aim of this study was to conduct a large-scale, 13-year nationwide cohort study based on claims data from the Taiwan National Health Insurance Research Database (NHIRD) in order to investigate the risk of ptosis among patients with DR. Additionally, we sought to investigate the impact of various co-factors of interest including certain medical comorbidities and their effect on the risk of developing ptosis.

## 2. Methods

### 2.1. Data source

The data for this study were based on the NHI program of Taiwan. Approximately 99.99% of Taiwanese citizens are currently enrolled in this healthcare program. Data analyzed for this retrospective cohort study were extracted from the Longitudinal Health Insurance Database (LHID), which is a randomized subset from the National Health Research Institute (NHRI) in Taiwan. The LHID contains around one million de-identified insurance beneficiary data that have been encrypted for privacy purposes. These data include: outpatient visits from each patient, hospital admissions, demographics, diagnoses, medical procedures and prescriptions. All diagnoses are recorded based on the International Classification of Diseases, 9th Revision, Clinical Modification (ICD-9-CM). Due to the de-identified and encrypted nature of each insurant data within the LHID database, informed consent was deemed not necessary by the regulations of the ethics committee of our institution. The study protocol was conducted according to the principles described in the Declaration of Helsinki. This study has been approved by the Research Ethics Committee at China Medical University Hospital, Taiwan (CMUH104-REC2-115-AR-4).

### 2.2. Study population

The study sample compromised of 9,494 patients with newly diagnosed DR (ICD-9-CM: 362.0, 362.01 and 362.02) that were recruited between January 1, 2000 and December 31, 2012. Patients with at least three outpatient visits for DR were defined as new cases and the first outpatient visit date for DR was set as the index date. Those with a diagnosis of DR prior to 2000 were excluded. Follow up would end when: the onset of ptosis (ICD-9-CM 374.3, 374.30, 374.31, and 374.32), date of death, data of withdrawal from the program or the end of the study period on December 31, 2013 occurred. Patients with at least two outpatient visits for ptosis, separated by at least 7 days, were defined as the endpoint. All study subjects were followed from the index date until the endpoint. Those without endpoint development were followed until the date of withdrawal from the program or the end of 2012, whichever occurred first. The study excluded patients whose index dates were not between 2000 and 2013, and had preexisting history of ptosis (ICD-9-CM 374.3, 374.30, 374.31, and 374.32) were excluded. Patients with viral hepatitis (ICD-9-CM code 070), cirrhosis (ICD-9-CM code 571, A347), interferon treatment, human immunodeficiency virus (HIV) infection (ICD-9-CM code 042–044, 795.8, V08), tuberculosis (ICD-9-CM code 010–012), syphilis (ICD-9-CM code 091.0, 095.4, 095.8), systemic malignancy (ICD-9-CM code 140–208), autoimmune diseases (ICD-9-CM code 135, 279.49, 283, 443, 571.42, 696, 710, 714, 715), chronic obstructive pulmonary disease (ICD-9-CM code 490–492, 494, 496), and asthma (ICD-9-CM code 493, 494) were also excluded. Subjects without outpatient visits for eye diseases were also excluded. Patients with co-factors of current smoking history (ICD-9-CM code V15.82, 305.1, 794.2), lipid metabolism disorders (ICD-9-CM code 272), and hypertension (HT) (ICD-9-CM code 401–405, A26) were included in the study. Controls were patients without history of DR (ICD-9-CM: 362.0, 362.01 and 362.02) and randomly selected from populations without histories of viral hepatitis, interferon treatment, HIV infection, tuberculosis, syphilis, or ptosis. Propensity score matching method was used to control for confounding factors and as part of this matching method, our control group was frequency-matched by age group (<20, 20–39, 40–64 and 65+ years old), gender, ophthalmologic outpatient department (OPD) before the index date, and index-year at a ratio 4:1. Only patients with at least one ophthalmology clinic visit before enrolling in the study were included. We then matched the ophthalmologic OPD visits between both groups.

### 2.3. Statistical analyses

*χ*^2^ testing was used to determine the difference in demographic characteristics between the DR cohort and comparison cohort from 2000 to 2012. *T*-test was employed for the difference of the mean OPD visit for ophthalmology between two cohorts. Continuous variables, such as age and follow-up time, were shown as mean and standard deviation (15) and analyzed by using the Wilcoxon rank sum test. The cumulative incidences of ptosis for both the DR and comparison cohorts were estimated by using the Kaplan–Meier method. The difference between the two curves was examined by using the log-rank test. A multivariable Cox model was adjusted for continuous age, gender, comorbidities, and OPD visits for ophthalmology before the index date. To minimize the effect of selection bias and control for potential confounding factors, a propensity score matching procedure was performed. The study group (patients with DR) and control group (patients without DR) were frequency-matched based on age group (<20, 20–39, 40–64 and 65+ years old), gender, ophthalmologic outpatient department (OPD) before the index date, and index-year at a ratio 4:1. Univariate and multivariable cox proportional regression analysis were used to measure the hazard ratio (HR) and 95% confidence interval (CI) to assess the association between DR and the risk of developing ptosis. The incidence density rate of ptosis (per-1,000 years) was calculated for DR cohort and comparison cohort. The risk of ptosis in the DR and comparison cohorts was stratified by age group, gender, and comorbidities, using Cox proportional hazard regression. SAS software (version 9.4 for Windows; SAS Institute, Cary, NC, United States) was used for all statistical analyses and creation of Kaplan–Meier survival curves. A two-sided *p* < 0.05 was considered statistically significant. The Wilcoxon rank-sum test was used for verification of average age and follow-up time. All the assumptions for the multivariate models were checked.

## 3. Results

### 3.1. Patient characteristics

Baseline characteristics of 9,494 DR patients and 37,976 cross-matched non-DR patients are tabulated in Table 1. The gender and age distributions were comparable in both groups according to the initial grouping design (Table 1). The follow-up time (year) was 6.93 ± 3.81 (mean ± SD) in DR group and 7.02 ± 3.83 in non-DR group (*p* = 0.04) (Table 1). As for comorbidities, the proportion of patients with lipid metabolism disorders or patients with hypertension were higher in the DR group than in the non-DR group (DR vs. non-DR: 17.9% vs. 6.52%, *p* < 0.01 for lipid metabolism disorders and 24.8% vs. 12.8%, *p* < 0.01 for hypertension, Table 1).

**Table 1:** Baseline characteristics of patients.

	Diabetic retinopathy (*n* = 9,494)		Non-diabetic retinopathy (*n* = 37,976)		*p*-value*
	*n*	%	*n*	%	
**Gender**					>0.99
Male	4,719	49.7	18,874	49.7	
Female	4,775	50.3	19,102	50.3	
**Age, years**					>0.99
<20	979	10.3	3,916	10.3	
20–39	3,372	35.5	13,488	35.5	
40–64	4,092	43.1	16,368	43.1	
≥65	1,051	11.1	4,204	11.1	
mean (Berg et al. 15)^a^	42.5 (17.5)	42.4 (17.5)	0.46
**Comorbidity**					
Current smoker	40	0.42	145	0.38	0.58
Lipid metabolism disorders	1,704	17.9	2,476	6.52	<.01
Hypertension	2,362	24.8	4,859	12.8	<.01
**Follow-up time, year** **^a^**	6.93 (3.81)	7.02 (3.83)	0.04

Diabetes mellitus included type 1 and type 2 diabetes mellitus.

^a^ Average age and follow-up time using *Wilcoxon rank-sum test* for verification.

* *p*-value using *χ*^2^ for the comparisons between with and without diabetic retinopathy.

### 3.2. Time to event analysis

**Figure 1:**
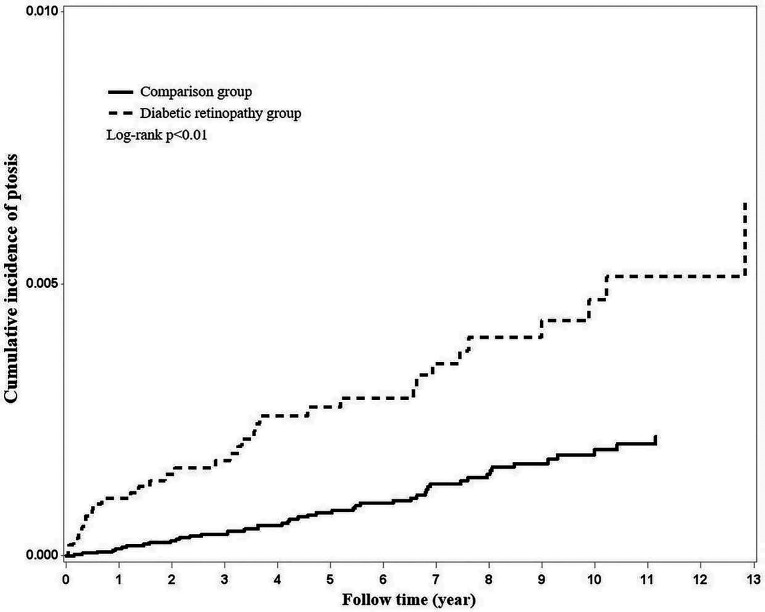
Using Kaplan–Meier survival statistics, it showed crude overall survival curves by with and without diabetic retinopathy. (log-rank *p* < 0.001).

**Figure 2:**
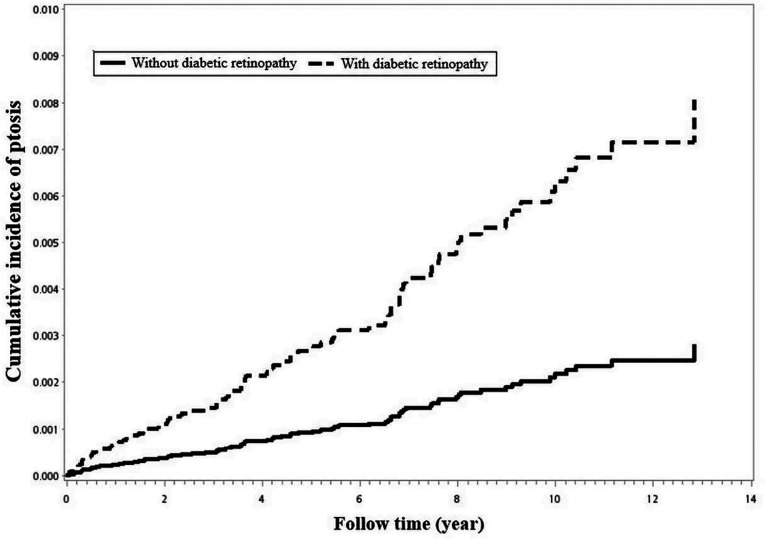
Using Kaplan–Meier survival statistics, it showed adjusted confounding factors survival curves by with and without diabetic retinopathy. (log-rank *p* < 0.001).

Using Kaplan–Meier survival statistics, crude overall survival curves of ptosis among DR and non-DR patients are shown in Figure 1. The cumulative incidence of ptosis increased with follow up years at faster rate among the DR group compared to the non-DR group (log-rank test *p* < 0.001, Figure 1). After adjusting for confounding factors, DR patients still demonstrated significantly greater cumulative incidence of ptosis (Kaplan–Meier analysis, log-rank test *p* < 0.001, Figure 2) compared to the control group. With Cox regression analysis, the DR group was found to have higher risk of developing ptosis (adjusted hazard ratio (HR) [95% CI]: 2.76 [1.74–4.38], *p* < 0.001) when compared to the control cohort (Table 2).

**Table 2:** Cox model measured hazard ratios and 95% confidence interval of ptosis associated with gender and age.

Variable	Ptosis			Crude HR (95% CI)	Adjusted HR (95% CI)
	Event	PY	IR		
**Diabetic retinopathy**					
No	47	266,927	0.17	**1(reference)**	**1(reference)**
Yes	33	65,860	0.50	2.84 (1.82–4.43)***	2.76 (1.74–4.38)***
**Gender**					
Female	31	161,415	0.19	**1(reference)**	**1(reference)**
Male	49	171,372	0.28	1.49 (0.95–2.34)	1.67 (1.06–2.63)*
**Age, years**					
<20	2	42,460	0.04	**1(reference)**	**1(reference)**
20–39	14	130,050	0.10	2.28 (0.52–10.0)	2.19 (0.49–9.66)
40–64	30	130,611	0.22	4.91 (1.17–20.5)*	4.71 (1.11–19.9)*
≥65	34	29,666	1.14	24.6 (5.90–102.7)***	20.7 (4.80–89.4)***
**Comorbidity**					
Current smoker					
No	0	739	0	**1(reference)**	**1(reference)**
Yes	80	332,048	0.24	–	–
Lipid metabolism disorders					
No	68	311,260	0.21	**1(reference)**	**1(reference)**
Yes	12	21,527	0.55	2.53 (1.36–4.70)**	0.84 (0.43–1.63)
Hypertension					
No	50	292,057	0.17	**1(reference)**	**1(reference)**
Yes	30	40,730	0.73	4.30 (2.73–6.78)***	1.43 (0.83–2.44)

PY, person-years; IR, incidence rate, per 1,000 person-years; HR, hazard ratio; CI, confidence interval.

HR adjusted for factors including gender, age and comorbidities of smoker, lipid metabolism disorders and hypertension.

* *p* < 0.05.

** *p* < 0.01,

*** *p* < 0.001.

### 3.3. The role of DR after stratification

When comparisons of the DR and non-DR groups were stratified by gender and age, significantly increased risks of developing ptosis patients with DR were found among the female gender [adjusted HR: 3.38 (1.89–6.04), *p* < 0.001, Table 3] and in adult patients of all age groups (adjusted HR: age 20–39, 3.07 [1.06–8.86], *p* < 0.05; age 40–64, 2.34 [1.08–5.06], *p* < 0.05; age ≥ 65, 2.53 [1.24–5.18], *p* < 0.05, Table 3). It should be noted that although the *p*-value for comparison of DR vs. non-DR group in male stratum was insignificant, the point estimate of DR group was >1, with adjusted hazard ratio being 1.98 (with 95% CI: 0.92–4.27). This overlaps with the 95% CI of female stratum. The implications of these results from our gender strata will be discussed later*.* When stratified by comorbidities, the association of DR was found to be stronger in non-smokers [adjusted HR: 2.76 (1.74–4.38), Table 3]. In terms of medical comorbidities of hyperlipidemia and hypertension, patients with DR had higher risk of developing ptosis compared to matched controls regardless of whether they had either of these two major medical comorbidities (Table 3).

**Table 3:** Incidence rate and hazard ratio of ptosis between with and without diabetic retinopathy stratified by gender, age and comorbidities.

Variable	Diabetic retinopathy						Crude HR (95% CI)	Adjusted HR (95% CI)
	No			Yes				
	Ptosis	PY	IR	Ptosis	PY	IR		
**Gender**								
Male	20	129,694	0.15	11	31,720	0.34	3.87 (2.14–6.99)***	1.98 (0.92–4.27)
Female	27	137,233	0.19	22	34,140	0.64	3.27 (1.86–5.74)***	3.38 (1.89–6.04)***
**Age, years**								
<20	0	33,991	0	2	8,469	0.23	–	–
20–39	8	103,732	0.07	6	26,317	0.22	2.96 (1.02–8.55)*	3.07 (1.06–8.86)*
40–64	18	105,269	0.17	12	25,343	0.47	2.79 (1.34–5.81)**	2.34 (1.08–5.06)*
≥65	21	23,935	0.87	13	5,731	2.26	2.57 (1.29–5.14)**	2.53 (1.24–5.18)*
**Comorbidity**								
Current smoker								
No	47	266,356	0.17	33	65,691	0.50	2.84 (1.82–4.43)***	2.76 (1.74–4.38)***
Yes	0	571	0	0	169	0	–	–
Lipid metabolism disorders								
No	46	254,586	0.18	22	56,674	0.38	2.14 (1.29–3.56)**	2.24 (1.34–3.76)**
Yes	1	12,341	0.08	11	9,186	1.19	15.0 (1.94–116.8)**	16.3 (2.10–127.4)**
Hypertension								
No	31	239,601	0.12	19	52,457	0.36	2.79 (1.58–4.95)***	3.21 (1.79–5.73)***
Yes	16	27,326	0.58	14	13,403	1.04	1.79 (0.87–3.67)	2.18 (1.05–4.53)*

PY, person-years; IR, incidence rate, per 1,000 person-years; HR, hazard ratio; CI, confidence interval.

Adjusted HR: multivariable analysis including for gender, age and comorbidities of current smoker, lipid metabolism disorders and hypertension.

– Unable to calculate because of there are few or no events in with and without diabetic retinopathy cohort.

* *p* < 0.05.

** *p* < 0.01.

*** *p* < 0.001.

### 3.4. Other possible clinical factors related to ptosis

In addition to DR, other clinical factors were statistically analyzed to assess their influence on the risk of ptosis through Cox regression analysis (Table 2). Older subjects (age ≥ 40) were found to have significantly increased risk of ptosis than their younger counterparts (adjusted HR for age 40–64: 4.71 [1.11–19.9], *p* < 0.05, adjusted HR for age ≥ 65: 20.7 (4.80–89.4), *p* < 0.001). Smoking, lipid metabolism disorders, and hypertension were found to be non-contributary towards the risks of developing ptosis after adjusting for other confounding factors (Table 2).

## 4. Discussion

To the best of our knowledge, this is the largest population-based retrospective cohort study that sought to evaluate the risk of developing ptosis among patients with (DR).

### 4.1. Novel findings

Our results showed that DR patients are at a higher risk of developing ptosis (adjusted hazard ratio (aHR) [95% CI]: 2.76 [1.74–4.38], *p* < 0.001) compared to non-DR patients. Adult patients of all age groups (adjusted HR: age 20–39, 3.07 [1.06–8.86], *p* < 0.05; age 40–64, 2.34 [1.08–5.06], *p* < 0.05; age ≥ 65, 2.53 [1.24–5.18], *p* < 0.05, Table 3) were found to increase the risk of developing ptosis among DR patients when compared to non-DR patients. Smoking, lipid metabolism disorders, and hypertension were found to be non-contributary towards the risks of developing ptosis after adjusting for confounding covariates.

### 4.2. Clinical implications

DM is a common medical condition and a contributing factor towards morbidity and mortality globally (16). Various ophthalmological sequala from DM include DR and cranial neuropathies (17). The primary intraocular manifestations for DM are DR and cataracts (4). Little is known however about the association between DR and ptosis. A small number of studies have observed tentative links between ptosis and DM or DR patients (18, 10, 19, 11, 20). Unfortunately, available studies have been limited to mostly studies of small power. The clinical implications from our study are through our contribution with the largest real-world data to date, the significant association between DR and the risk of developing ptosis. These findings are relevant clinically as better understanding of the nature and patterns of comorbidities among diabetic patients may provide valuable insights for managing such patients with multiple conditions in everyday clinical practice.

### 4.3. Comparisons to other studies

Initial understanding of DM was that it is a multisystem disorder that involves complications with neurological and vascular components. DR is one of the most common diabetic complications and underlining mechanism involves pathological alterations of the blood vessels of the retina (21). Diabetes can also potentially cause development of neurological conditions like ptosis secondary to hyperglycemia-induced damage to nerve cells and neuronal ischemic change (22). The relationship between ptosis and DM have been described in various literature but are mostly limited to small studies. Two studies of note will be discussed below.

The first study was by Bosco et al., which was a retrospective cohort study that recruited 162 ptosis patients and 128 control participants in order to investigate the prevalence of diabetes among their study cohorts (20). Their result showed high prevalence of diabetes among ptosis patients, with diabetes being diagnosed in 36 out of the 162 (0.27%) of their ptosis participants. After adjusting for cofounders, statistical significance was found between ptosis and diabetes when compared to the control group (*p* < 0.001). Furthermore, smoking status (*p* = 0.25), high blood pressure (*p* = 0.79) and serum lipid (*p* = 0.79) were found to be noncontributory towards developing ptosis when compared to the control group. This complimented our findings. We also demonstrated the non-contributory nature of high lipid levels and smoking status towards the relationship between ptosis and DR.

In a second study of note on this topic, the Korea National Health and Nutrition Examination Survey (KNHANES) by Moon et al. was a cross-sectional cohort study that compromised of 13,461 participants (11). Their results showed diabetes to be significantly associated with ptosis and is an independent risk factor for ptosis after adjustment for confounding variables. Their results complemented our own in some aspects with some major differences. One major difference was with regards to the influence of the comorbidities of hyperlipidemia and HT on ptosis risk. Moon et al. demonstrated hyperlipidemia and HT to be risk factors for developing ptosis among their diabetic study participants (11). This is in contrast to ours and Bosco et al., which showed no significant association (20). It should be noted in Moon et al. that the association between hyperlipidemia and ptosis was specifically made with regards to their triglyceride levels (*p* < 0.001) only. Other lipid levels including LDL-C (*p* = 0.956) and cholesterol (*p* = 0.847) from the Moon et al. study was found to be non-contributory (11). Furthermore in Moon et al., the link between triglyceride level was established with ptosis after adjustment with age and sex only. However, after application of multiple regression analysis with other co-founding variables being excluded, triglyceride levels from Moon et al. were then found to be non-significant (*p* = 0.049) towards ptosis risk (11). This implies that the statistical strength of ptosis and hyperlipidemia to be weak and possibly requires further investigations to validate. On another note, one of the limitations of our study was that we defined our hyperlipidemia disorder based on ICD diagnosis. This meant that it is unknown which type of lipid levels were abnormal among our participants and so direct comparison between our study and Moon et al. cannot be accurately made.

Another major difference made by Moon et al. compared to our study was their association of ptosis among diabetic patients with the comorbidity of hypertension (11). However, again, as mentioned earlier, this relationship was only seen with ptosis when adjusted for age and sex (*p* < 0.001). When other cofounding variables were excluded secondary to multiple regression analysis, hypertension was subsequently then found to be not significantly associated with ptosis (*p* = 0.173) in the Moon et al. study (11). This again implied the statistical weakness of this relationship and future studies are again required to validate this association.

Lastly, in terms of gender, Moon et al. found that male diabetic participants have increased risk of ptosis (11). Unfortunately, we were unable to provide definite conclusions from our results in terms of gender. The reasons were as follows: the aHR in male stratum was still greater than 1 (1.98), which means that the possibility of DR-male having a higher risk for ptosis than non-DR-male still exists, even though the *p*-value is greater than 0.05. A *p*-value >0.05 does not mean there is no difference, merely that we found no evidence for an effect. *p*-value is a composite which depends not only on the size of an effect but also on how precisely the effect has been estimated (its standard error). Therefore, we were unable to tell whether the difference in *p*-values arose because there was no effect in male group or if there was an effect, perhaps even one of similar size to that in female group, but was less precisely estimated. Thus, the influence of gender variable or its interaction with DR on the development of ptosis should be further confirmed in future studies. However, if there does exist gender-specific interactions with ptosis risk, we hypothesize that certain gender related alleles may have played a role. Examples of such genes include the u-opioid receptor gene (OPRM1). This gene has been shown to increase the severity of diabetic neuropathy among females but confers a protective effect among male genders (23).

### 4.4. Strengths and limitations

There are several strengths from this study. Firstly, it is based off the Taiwanese Longitudinal Health Insurance Database (LHID), which is a randomized sampled subset of a national population cohort. This ensures that ours is a representative nation-wide sample of DR patients from the Taiwanese population. Furthermore, to the best of our knowledge, ours is the largest population-based study to date that evaluated the association between ptosis and DR. These characteristics as well as our long follow up period and having a well-matched control cohort further ensures the reliability of our results.

Our study however has a few limitations. Firstly, the retrospective design of our study inherently limits any conclusions made to be based on associations and not causality. Furthermore, the ICD coding-based diagnosis of our study means that the severity of DR and smoking duration among our study participants was unknown. The severity of DR is important as more advanced stages of DR or proliferative DR (PDR) are usually associated with prolonged duration of diabetes. Other studies have reported independent effects of diabetes duration on the risk of diabetic complications (24). Possible etiological explanations include uncontrolled hyperglycemia causing irreversible vascular damage which would increase the risk of complications (25). Therefore, it is essential in future studies to include “DR severity” and “diabetes duration” in the statistical analysis to evaluate for independent effect of these factors on the development of ptosis. A point to note related to this is that our control group excluded DR but did not exclude diabetes. This is perhaps another confounding source as we were unable to isolate the effect of diabetes from our control group. Therefore, some valid questions can be raised about the comparability of our control group is to the study group as the diabetes severity among our participants are unknown in both the respective control and study group.

Another limitation of ICD-coding is that smoking duration cannot be accurately represented either. Therefore, any dose-dependent association between smoking, DR, and ptosis cannot be drawn from our results. Future studies of prospective nature could include taking down details of first diagnosis of diabetes as well as a comprehensive smoking survey to be collected at each visit. Information gathered at each visit could include: number of years smoked, cigarette smoked per day, current vs. former smoking status and the final quit date. Thirdly, the possibility of misdiagnosis of DR with non-DR ischemic retinopathy cannot be definitively excluded from the database. Non-DR ischemic retinopathy are not uncommonly seen among diabetic patients, as the prevalence of nondiabetic retinal pathologies have been reported in one study to range from 4.5%–6.5% (26). However, despite the possibility of misdiagnosis, there are ICD codes specific for DR and other ischemic retinopathy which should limit any misclassification bias. Another point to note was that we did not obtain data on which subclassification of ptosis would be more affected among DR patients. However, it should be noted even if we had included this, there are still limitations associated with it as well. Primarily, the source of the ICD diagnosis (whether for ptosis subclassification or for DR) would be another potential confounding factor. It is unknown whether the ICD code was obtained from a general practitioner (GP) or from a specialist. However, exploring which ptosis subclassification would be affected could be interesting to explore in future studies.

Additionally, there are some inherent limitations present in electronic health records registry (EHR) like the Taiwanese LHIRD that deserves some discussion. One issue is with regards to the issue of representativeness. Firstly, in spite of our numerous study design that have attempted to ensure the validity of our study and control groups, the inherent nature of EHR means that our results may have overrepresented participants with chronic medical history within our study's set time period. This potentially introduces bias where those with more medical encounters are more prone to be diagnosed with medical conditions (27). Furthermore, our database would have underrepresented participants who for whatever reason, are not as compliant with follow ups or have poorer access to medical service. This is a form of selection bias that is related to a discordancy between our study population and the real-world target population. In a study by Romo et al. they showed that those with increased medical encounters tend to be: female, unemployed, white ethnicity and of the lower social-economic status (28). Secondly, our EHR data may have missing or incorrect clinical information for each of our study participants. This has implications in terms of selection or information bias as we assume that those without the ICD diagnostic code of uveitis or DR were absent of the disease of interest when in fact it may have been a clerical mistake. However, due to the de-identified nature of this database, we lack the ability to individually confirm each of our patient's clinical information. Overall, despite these limitations, we have tried to minimize these effects through our large sample size, setting our time period of recruitment to be over 12 years in duration and also frequency matching based on OPD visits. Future studies could include prospective nature of which each individual participants clinical trajectories and information can be verified from each medical visit.

Another point to note is that we did not exclude any patients based on underlining neurological conditions. It is possible that underlining neurological conditions could have an effect on our results as ptosis frequently manifest in association with other neurological disorders such as stroke (29).

One other point to note was that our study was composed of an entirely ethnic Asian Taiwanese population. This would have issues of generalizability to other studies with different ethnic work up. Patients of Asian ethnicity have been shown to be more predisposed to diabetic complications than compared to their Caucasians counterparts (30). How this translates towards actual risk of developing ptosis among DR patients is still unknown.

Another limitation is with regards to the existing statistical analysis from our study that was applied to the variable of gender. As part of our analysis of different strata, the risk of DR for ptosis were found to be higher for some subgroups stratified by some variables such as age and smoking status. The risk of ptosis was predominantly observed in adult age and non-smoker groups when compared to their counterparts. But for other co-morbidities, the risk of DR for ptosis were confirmed irrespective of the presence of these co-morbidities. As for the variable of gender, although the higher risk of DR for ptosis was not confirmed in male stratum like in the female stratum, the conclusion that female DR patients were more inclined to have ptosis than male could not be definitely made. The reasons were outlined earlier and include the fact that the aHR in male stratum was greater than 1 (1.98). This means that there exists the possibility of our DR-male having higher risk for ptosis compared to the non-DR-male. However, any conclusion that can be drawn from this is limited as the *p*-value obtained was greater than 0.05. Our lack of conclusion on the factor of gender for ptosis risk is concerning as studies like Moon et al. have shown male diabetic patients to be more predisposed to ptosis risk (11). Therefore, the influence of the gender variable and its potential interaction with DR on the development of ptosis should be further confirmed in future studies and could possibly include the test of heterogeneity.

## 5. Conclusion

In conclusion, our large scale, population-based, 13-year retrospective cohort study have demonstrated DR patients to possess significantly increased risk of developing ptosis complications compared to patients without DR. Co-factors of nonsmoker and adult patients has also been shown to increase the risk of ptosis. The results of our study are relevant because they demonstrate with the largest data to date, the influence of DR on the risk of ptosis. Better understanding of comorbidities like ptosis among DM or DR patients may facilitate clinicians to better forecast such complications whenever they may arise and tailor therapeutic interventions specific for such patients. Before ptosis surgery, the possibility of underlying diabetes or DR should be also scrutinized and treated properly to avoid undesirable postoperative dissension.

## Data Availability

The raw data supporting the conclusions of this article will be made available by the authors, without undue reservation.
